# GMD-YOLO: A Dual-Modality Framework with Multi-Scale Enhancement and Adaptive Fusion for PV Fault Detection

**DOI:** 10.3390/s26113394

**Published:** 2026-05-27

**Authors:** Zhichao Lin, Xiuling Wang, Yuyang Guo

**Affiliations:** 1 College of Information Engineering, Inner Mongolia University of Technology, Hohhot 010080, China; 20241100120@imut.edu.cn (Z.L.);; 2 Inner Mongolia Key Laboratory of Perceptive Technology and Intelligent Systems, Hohhot 010080, China

**Keywords:** photovoltaic modules, dual-modality detection, visible–infrared fusion, YOLO11, object detection, Shape-IoU

## Abstract

Photovoltaic (PV) module faults, such as hotspots, diode short circuits, occlusions, and shadows, degrade power generation efficiency and safety. Existing manual inspection and single-modality methods show limited robustness under complex conditions, especially with illumination variations and weak thermal responses, while most deep learning approaches fail to exploit the complementarity of visible and infrared modalities. To address this issue, a dual-modality visible–infrared fusion framework based on YOLO11 is proposed, integrating a multi-scale pyramid pooling and dilated convolution module (MSPPD), a gradient-aware fusion module (GAFusion), and a dynamic convolution and element-wise scaling detection head (Detect-DEhead). GAFusion enhances cross-modal structural consistency and reduces feature misalignment and information loss during fusion by introducing gradient-aware feature interaction. Shape-IoU loss is employed to improve localization accuracy. The proposed method improves mean average precision (mAP)@0.5 from 86.7% to 88.1%, while reducing parameters, computational cost, and model size from 4.3 M to 3.7 M, 11.42 GFLOPs to 9.37 GFLOPs, and 9.1 MB to 7.9 MB, respectively. With Shape-IoU, mAP@0.5 reaches 88.4%, and recall increases from 78.5% to 84.9%. Experiments on the FLIR Thermal dataset achieve gains of 2.2%, 1.6%, and 2.7% in precision, recall, and mAP@0.5. The method achieves an effective trade-off between accuracy and efficiency for intelligent PV module inspection.

## 1. Introduction

Fault detection of photovoltaic (PV) modules is a critical component for ensuring the safe, stable, and efficient operation of PV power plants. Its accuracy and robustness directly affect energy utilization efficiency and operation and maintenance cost control. With the continuous increase in installed PV capacity, modules are prone to various faults during long-term operation, including hotspots, diode short circuits, occlusions, and shadows. These faults not only reduce power generation efficiency but may also lead to potential safety hazards, such as localized overheating, module damage, and even fire risks [1]. Particularly in centralized and distributed PV power plants, the large number of modules and complex operating environments make traditional methods relying on manual inspection or experience-based judgment insufficient to meet the demands of high-frequency, large-scale, and fine-grained operation and maintenance. Manual inspection is not only time-consuming and labor-intensive but is also susceptible to subjective factors, often resulting in missed detections and false alarms. In practical drone-based PV inspection, the onboard computing capability, battery capacity, and communication bandwidth are inherently limited. Therefore, fault detection models are required to achieve a balance between detection accuracy, computational complexity, and real-time inference efficiency. Lightweight edge-deployable detection frameworks can effectively reduce maintenance costs, energy consumption, and data transmission burden while improving inspection efficiency in large-scale PV power plants.

Existing fault detection methods for photovoltaic modules based on a single modality typically rely on either visible or infrared imaging. Visible images are advantageous in capturing surface textures, structural details, and occlusions. However, under conditions such as strong illumination variations, surface reflections, or shadows, subtle faults such as hotspots and shadow-related defects tend to suffer from feature degradation. In contrast, infrared images can effectively reflect thermal radiation differences between faulty and normal regions, showing strong responses to thermally anomalous faults such as hotspots and diode short circuits. Nevertheless, their capability to distinguish low thermal contrast features, such as occlusions and shadows, remains limited [2,3]. As a result, single-modality methods struggle to provide stable and comprehensive feature representations in complex environments, leading to prominent issues of missed detections and false alarms, particularly for weak-contrast or small targets such as shadows and occlusions [4].

Hotspot faults typically manifest as localized abnormally high-temperature regions on PV modules, which form distinct thermal signatures in infrared images but may be nearly invisible in visible images, particularly under strong illumination reflection or module aging conditions. Diode short-circuit faults lead to localized temperature increases, where infrared imaging can effectively capture thermal diffusion characteristics, while visible images only exhibit subtle appearance anomalies. Occlusion faults mainly affect the surface illumination of the module, appearing clearly in visible images, whereas in infrared images, they are often confused with local environmental temperature variations. Shadow faults are characterized by locally reduced illumination, which is easily identifiable in visible images but exhibits weak thermal contrast and indistinct features in the infrared modality. Therefore, different fault types exhibit significant representation discrepancies across the two modalities, which provides a natural advantage for dual-modality fusion but also increases the complexity of feature alignment and integration [5,6]. Moreover, visible and infrared modalities exhibit an inherent semantic gap, since visible images mainly capture texture, color, and structural information, whereas infrared images primarily reflect thermal radiation distributions [7]. As a result, direct feature concatenation may fail to establish effective semantic correspondence between modalities, particularly under weak thermal response conditions [8], leading to the loss of fine-grained edge details and degraded representation of subtle faults such as shadows and occlusions.

To overcome the performance limitations of single-modality detection, dual-modality fusion has emerged as an important direction for photovoltaic module fault detection. By jointly leveraging visible and infrared information, it enables the simultaneous capture of thermal anomaly features of hotspots and diode short circuits, as well as visible texture features of occlusions and shadows, thereby achieving comprehensive coverage of multiple fault types. In recent years, multimodal object detection techniques have advanced rapidly. Strategies such as cross-modality alignment, noise suppression, and feature reweighting have effectively mitigated the degradation of single-modality information, providing strong technical support for the all-time and all-weather intelligent inspection of PV modules [9,10,11]. To systematically evaluate the contribution of dual-modality information to PV fault detection, this study further conducts a comparative analysis between single-modality and dual-modality models across different fault categories, thereby validating the effectiveness of the proposed multimodal fusion strategy.

In dual-modality fusion strategies, existing methods can be broadly categorized into pixel-level fusion, feature-level fusion, and decision-level fusion. Pixel-level fusion integrates multimodal images through channel concatenation, weighted summation, or simple mapping operations [12,13]. Although this approach is computationally efficient and easy to implement, it is highly sensitive to differences in modality intensity distributions and low-level noise, which may weaken critical features and particularly fail to preserve fine-grained information such as hotspots and shadows. Decision-level fusion performs integration at the level of independent detection outputs via confidence weighting, voting schemes, or rule-based constraints [14,15]. While offering high flexibility, it operates at the final prediction stage and thus cannot fully exploit deep semantic correlations between modalities, resulting in limited discriminative ability for fine-grained faults such as micro-hotspots, occlusions, and local shadows. In contrast, feature-level fusion enables joint modeling and the dynamic interaction of multimodal features within intermediate network layers. It not only alleviates the noise amplification problem of pixel-level fusion but also compensates for the insufficient information exploitation of decision-level fusion, making it one of the most promising fusion paradigms in YOLO-based dual-modality detection frameworks [15,16,17].

In feature-level fusion, three key issues should be addressed. First is its multi-scale feature extraction capability. Faults in photovoltaic modules exhibit significant scale variations. For example, hotspots typically appear as small localized high-temperature regions, whereas shadows may cover large areas. Therefore, the network is required to possess strong multi-scale feature modeling capability. The second issue is modality response discrepancy modeling. Visible and infrared modalities respond differently to the same fault, and the fusion module should adaptively select informative features through dynamic weighting mechanisms. Finally, spatial alignment. Visible and infrared images often suffer from viewpoint misalignment and pixel-level shifts; without effective alignment, feature confusion may occur, thereby degrading detection performance [15,16,17].

Numerous studies have demonstrated that well-designed feature-level fusion modules can notably improve dual-modality detection performance. For instance, Gu et al. [16] proposed a dynamic gating-based feature fusion method that adaptively balances modality contributions and effectively alleviates modality mismatch. EAFF-Net [13] enhances infrared and visible feature interaction via an efficient attention mechanism, achieving improved detection accuracy while maintaining computational efficiency. MOD-YOLO [15] introduces a cross-stage Transformer fusion module to enable the deep semantic alignment of multimodal features. MBUDet [17] addresses spatial misalignment in dual-modality data through feature-level alignment strategies, improving robustness in complex scenarios. Despite these advances in feature fusion and cross-modal interaction, several limitations remain. Most existing methods are designed for general scenarios and lack explicit modeling of modality discrepancies for specific fault types. In addition, many approaches fail to adequately account for modality-specific response inconsistencies during fusion, which may weaken critical features. Furthermore, detection performance for small objects and low-contrast defects remains limited.

Compared with earlier YOLO versions, such as YOLOv8 and YOLOv10, YOLO11 introduces an optimized architecture that improves feature representation and extraction efficiency. Its enhanced backbone and feature fusion design provide stronger multi-scale modeling capability, which is particularly beneficial for dual-modality PV fault detection tasks involving small targets, weak thermal signatures, and complex backgrounds. In YOLO-based dual-modality detection, Xue et al. [6] introduced a dynamic convolution module into YOLO11 and proposed the YOLO-ALDS framework, improving representation of fine-grained defects and blurred boundaries. Liang et al. [9] performed feature-level fusion of YOLO with millimeter-wave radar and camera data, significantly enhancing performance under complex operating conditions. In applications such as underwater structure inspection and industrial crack detection, YOLO-based multimodal methods combined with image enhancement have demonstrated strong robustness under extreme conditions [18]. Recent studies on lightweight yet high-accuracy multimodal detection, such as EsophagealDET [19], the CMIFDF framework [20], and RGB-T detection methods targeting extreme modality imbalance [21], further promote the engineering deployment of dual-modality models in complex environments. In particular, lightweight multi-scale feature enhancement networks based on YOLO11 have shown strong performance in small-object and occlusion scenarios, providing valuable insights for detecting subtle PV faults such as hotspots and shadows [22].

Despite substantial progress in feature extraction, fusion strategies, lightweight design, and complex scene adaptation of YOLO-based dual-modality detection methods, several limitations remain for photovoltaic fault detection. First, existing multi-scale feature aggregation modules have limited capability in jointly modeling strongly responsive faults (e.g., hotspots) and low-contrast defects (e.g., shadows). In practical PV inspection scenarios, hotspots and diode short circuits usually appear as localized thermal anomalies requiring strong sensitivity to fine-grained details, whereas shadows and occlusions often exhibit weak thermal contrast and irregular spatial distributions, making stable feature extraction and accurate localization more difficult. Second, general-purpose detection heads lack explicit modeling of modality discrepancies and geometric variations, leading to unstable classification and localization performance under complex illumination, partial occlusion, weak thermal response, and modality imbalance. Third, most existing fusion strategies rely on unified or static designs, making it difficult to adaptively emphasize informative features from either visible or infrared modalities for different fault types. Therefore, systematically optimizing multi-scale contextual modeling, modality-aware detection, and feature fusion mechanisms within a YOLO11 framework tailored to the dual-modality characteristics of PV faults remains a critical open challenge. The main contributions of this work can be summarized as follows:

1. To address the challenges of large-scale variation and low contrast in photovoltaic fault detection, a dual-modality detection framework is proposed for four typical fault types, namely, hotspots, diode short circuits, occlusions, and shadows. An enhanced multi-scale contextual aggregation module is introduced to effectively integrate features from different receptive fields, improving the representational capability for small objects and low-contrast targets.
2. A modality-aware dynamic detection head is designed to adaptively adjust the contribution of different modalities, enabling task-specific feature enhancement. Specifically, infrared features are emphasized for thermally anomalous faults such as hotspots and diode short circuits, whereas visible features are strengthened for appearance-related faults such as occlusions and shadows, thereby improving classification accuracy and detection stability.
3. An adaptive dual-modality feature fusion mechanism is proposed to achieve deep semantic alignment and dynamic interaction between heterogeneous modalities at the feature level, effectively mitigating distribution discrepancies and spatial misalignment while improving robustness in complex scenarios.
4. The conventional CIoU loss function is replaced by a Shape-IoU loss function for bounding box regression optimization [23]. By jointly considering object shape, aspect ratio variation, and spatial distribution, the proposed loss improves localization robustness for elongated and irregular defects such as shadows and occlusions while enhancing convergence performance without increasing model complexity.

Extensive experimental results demonstrate that the proposed method consistently improves performance across four typical PV fault detection tasks. In particular, it achieves superior accuracy and robustness under challenging conditions, such as the presence of small objects, low contrast, and occlusions, outperforming both single-modality baselines and representative state-of-the-art dual-modality methods, such as EAFF-Net and MOD-YOLO. This study provides a reliable solution for intelligent PV inspection systems and offers valuable insights for multimodal small object detection, dynamic modality weighting, and lightweight network design.

## 2. Materials and Methods

### 2.1. Data

#### 2.1.1. Data Acquisition

The dataset used in this study was collected through UAV-based inspection of large-scale centralized PV power plants. To comprehensively capture both the appearance information and thermal characteristics of PV modules under different operating conditions, a DJI Matrice 300 RTK industrial-grade UAV platform (DJI Innovation Technology Co., Ltd., Shenzhen, China) equipped with a Zenmuse H20T hybrid sensor system (DJI Innovation Technology Co., Ltd., Shenzhen, China) was employed for synchronized visible–infrared data acquisition. This setup ensures consistency in equipment and imaging parameters, thereby enabling the construction of a high-quality dual-modality PV defect detection dataset. Specifically, the visible-light camera integrated in the Zenmuse H20T sensor system provides a resolution of 5184 × 3888, offering rich structural and textural details, while the thermal infrared sensor operates at a resolution of 640 × 512, effectively capturing thermal anomalies in PV modules. During data acquisition, the UAV performed systematic inspections of PV arrays under stable flight conditions. In practical UAV-based photovoltaic inspection scenarios, visible and infrared images may exhibit significant cross-modal discrepancies caused by differences in imaging mechanisms, flight altitude variations, and viewpoint changes. These factors can lead to scale inconsistency, spatial offset, and structural misalignment between modalities, thereby affecting the reliability of subsequent multimodal fusion and fault detection. To alleviate these issues, the visible and infrared sensors were mounted on a unified UAV platform with fixed relative positions, and synchronized acquisition was performed during flight inspections. Meanwhile, millisecond-level synchronization, three-axis mechanical stabilization, and consistent flight trajectories were adopted to minimize viewpoint deviations and motion disturbances, ensuring precise spatial–temporal correspondence between modalities. The flight altitude was maintained between 20 and 30 m, achieving a balance between operational safety and sufficient imaging resolution. In the data preprocessing stage, static images were extracted from inspection videos using fixed-interval frame sampling. A rigorous quality control and registration process was then applied to remove low-quality samples, such as blurred or redundant images, ensuring the validity and alignment accuracy of the multimodal data. Considering the input size requirements of deep learning models and GPU memory constraints, all images were spatially normalized. Specifically, high-resolution visible images were down sampled to 640 × 640, while infrared images were cropped to match the same resolution.

Ultimately, a total of 1747 high-quality pairs of visible and infrared images were obtained. Each pair corresponds to the same PV module region captured at the same time and from the same viewpoint, jointly representing the target from both visual and thermal modalities. The resulting dataset exhibits strong spatial–temporal alignment and complementary information in terms of structural details and thermal characteristics, providing a reliable foundation for subsequent multimodal feature fusion and PV defect detection. The dataset was further divided and utilized for model training, validation, and testing.

#### 2.1.2. Fault Types and Analysis

**Figure 1:**
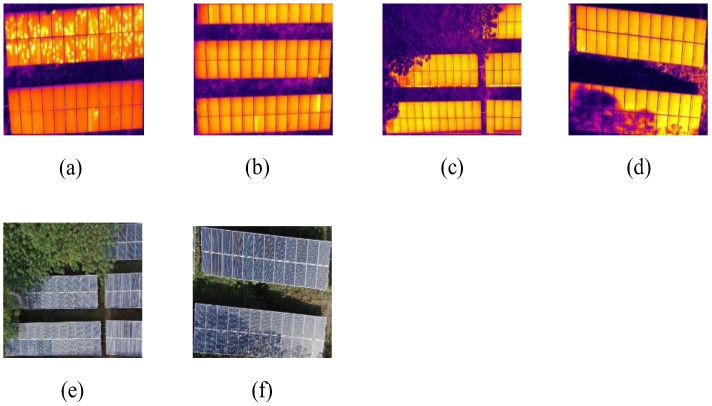
(**a**) Hotspot fault in thermal infrared image; (**b**) diode short-circuit fault in thermal infrared image; (**c**) occlusion fault in thermal infrared image; (**d**) shadow fault in thermal infrared image; (**e**) occlusion fault in visible image; (**f**) shadow fault in visible image.

After analyzing the collected PV images, four typical types of faults were identified in the PV power plant, namely, hotspots, diode short circuits, shadows, and occlusions. Representative images of different fault types are shown in Figure 1.

Hotspot faults typically appear as localized high-temperature regions in thermal infrared images, forming bright areas, while exhibiting little to no distinguishable appearance features in visible images. These faults are often caused by micro-cracks, local damage, or manufacturing defects in solar cells, and are characterized by a small target size and scattered distribution. As a result, they are easily submerged in background noise during detection, increasing the risk of missed detections. Diode short-circuit faults usually occur in the junction box on the back of PV modules. In thermal infrared images, they manifest as regular-shaped regions with significantly elevated temperatures, often appearing as rectangular or strip-like hotspots [24], whereas they are nearly indistinguishable in visible images. This type of fault is characterized by strong thermal signatures but weak visible semantics, posing higher requirements for multimodal feature fusion. Shadow faults are mainly caused by external objects such as trees or buildings casting shadows onto the surface of PV modules. They typically appear as irregular dark regions in visible images, while in thermal infrared images, they may exhibit uneven temperature distributions. Due to blurred boundaries, varying shapes, and sensitivity to illumination conditions, shadow faults are easily confused with background regions, increasing the difficulty of feature extraction and object localization. Occlusion faults are caused by physical objects (e.g., dust accumulation, bird droppings, or foreign materials) partially covering the surface of PV modules. In visible images, they usually appear as irregular regions with distinct texture or color differences from the background, whereas in thermal infrared images, they often exhibit weak or ambiguous thermal responses. This type of fault presents clear appearance cues but limited thermal contrast, which may lead to misclassification or omission when relying on a single modality.

#### 2.1.3. Dataset Construction

For the four fault types—hotspots, diode short circuits, occlusions, and shadows—manual annotation was performed on both visible and thermal infrared images to ensure consistency in object categories and spatial locations across modalities. Standard object detection annotation tools (e.g., LabelImg) were used to generate corresponding label files in YOLO format, satisfying the input requirements of deep learning-based detection models.

During dataset construction, all 1747 image pairs were divided into training, validation, and test sets with a ratio of 8:1:1 to ensure sufficient training and objective evaluation. The training set was used for model parameter learning, the validation set for model tuning and overfitting prevention, and the test set for final performance evaluation.

The dataset covers diverse complex backgrounds and fault targets at different scales, effectively reflecting real-world UAV inspection scenarios in PV power plants and providing reliable support for evaluating dual-modality detection models.

### 2.2. Object Detection Network

In recent years, the YOLO (You Only Look Once) series has become a representative one-stage object detection framework, continuously achieving a balance between detection accuracy and inference efficiency [25]. Starting from YOLOv5, the series established a foundation for lightweight real-time detection through highly engineered designs and mature training strategies. Subsequently, YOLOv6 and YOLOv7 further optimized network structures and feature reuse mechanisms, leading to notable improvements in performance and stability.

YOLOv8 and YOLOv9 introduced decoupled detection heads and more efficient feature fusion strategies, effectively alleviating feature conflicts between classification and regression tasks and enhancing model generalization. More recent versions, from YOLOv10 to YOLOv13, focus on end-to-end efficiency, structural consistency, and high-level semantic modeling, showing strong potential in complex scene understanding. However, as model complexity increases, deployment costs on resource-constrained platforms also rise.

In comparison, YOLO11 achieves a better balance among detection performance, model size, and computational efficiency [26]. Its clear modular design and well-defined multi-scale feature hierarchy provide a solid foundation for feature-level fusion and multimodal extensions.

Notably, YOLO11 provides a series of model variants with different scales, including YOLO11n, YOLO11s, YOLO11m, YOLO11l, and YOLO11x, which offer flexible trade-offs between accuracy and computational cost. Smaller models (e.g., YOLO11n and YOLO11s) are lightweight and suitable for resource-constrained scenarios, while larger models (e.g., YOLO11l and YOLO11x) achieve higher accuracy at the expense of increased parameters and computational complexity.

Considering the requirements of UAV-based deployment and real-time processing, as well as the limitations of computational resources, YOLO11n is selected as the baseline model in this study and further optimized for visible–thermal infrared PV fault detection tasks.

### 2.3. Proposed GMD-YOLO Model

**Figure 2:**
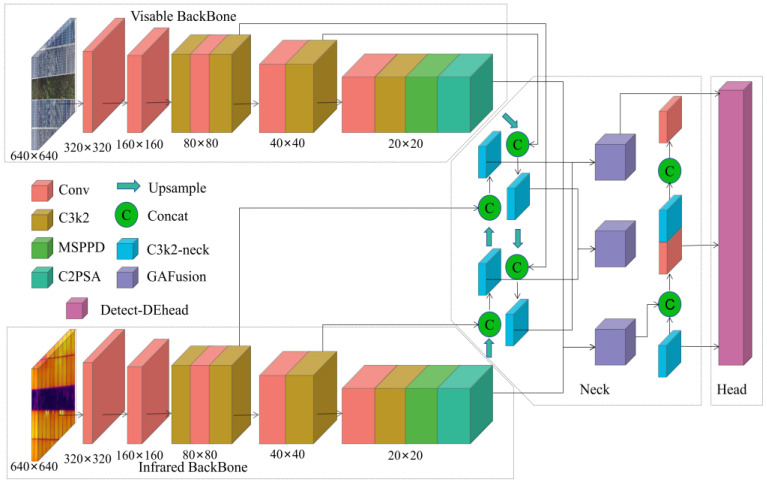
Architecture of the proposed GMD-YOLO dual-modality framework.

YOLO11, as a recent generation in the YOLO series, inherits the design philosophy of a lightweight backbone, a feature pyramid-based neck, and a decoupled detection head, achieving a favorable balance between detection accuracy and inference efficiency [27]. Its backbone mainly consists of convolutional layers and C3k2 modules for efficient extraction of multi-level semantic features. At the high-level feature extraction stage, YOLO11 employs the Spatial Pyramid Pooling Fast (SPPF) module to enlarge the receptive field and incorporate multi-scale contextual information. The neck adopts a PAN/FPN structure to perform cross-scale feature fusion through upsampling and feature concatenation, while the head utilizes a standard detection head for object localization and classification. To address the challenges in UAV-based dual-modality PV fault detection, such as dense small targets, complex backgrounds, and insufficient utilization of cross-modal information, this study introduces targeted modifications to the original YOLO11 architecture. In addition, the mid-to-late fusion strategy in YOLOv11-RGBT proposed by Wan et al. [28] is adopted as a reference. The overall architecture of the proposed GMD-YOLO dual-modality framework is illustrated in Figure 2.

First, at the high-level feature extraction stage of the backbone, the original SPPF module is replaced with the proposed multi-scale pyramid pooling and dilated convolution (MSPPD) module. This modification enhances multi-scale feature modeling and feature representation without altering the overall network structure, thereby improving the backbone’s ability to capture fine-grained defect features. Second, during the feature fusion stage in the neck, the original YOLO11 employs a simple concatenation (Concat) operation to fuse features from different scales. In this study, the mid-to-late fusion structure is replaced with the proposed gradient-aware fusion (GAFusion) module. This transformation enables the fusion process between visible and infrared features to evolve from passive aggregation to active interaction, thereby more effectively exploiting the complementary advantages of texture information and thermal radiation information across modalities. Finally, in the head stage, the original detection head of YOLO11 is replaced with the improved dynamic convolution and element-wise scaling detection head (Detect-DEhead). This modification optimizes the regression and classification tasks through structural decoupling and dynamic feature modeling, significantly improving the localization accuracy and recognition capability for small-scale and irregular PV fault targets.

Through the above module-level replacements and structural optimizations, the proposed method enhances feature representation and detection performance for dual-modality PV fault detection while preserving the lightweight design philosophy of YOLO11n. The detailed structure and working mechanisms of each module will be elaborated on in the following sections.

### 2.4. Multi-Scale Pyramid Pooling and Dilated Convolution Module

**Figure 3:**
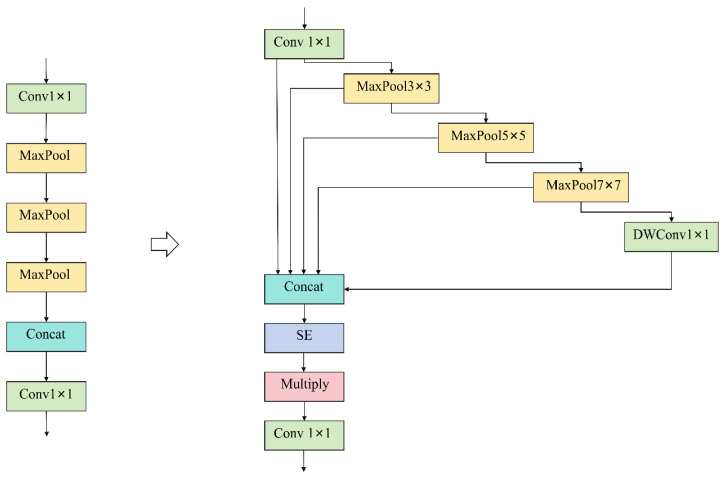
Comparison between the SPPF (**left**) and MSPPD (**right**) modules.

The core advantage of replacing the SPPF module with the proposed MSPPD lies in its ability to significantly enhance multi-scale contextual modeling, feature diversity, and channel-wise adaptive modulation while maintaining a lightweight design, as illustrated in Figure 3.

The conventional SPPF adopts a single-scale cascaded max-pooling operation (typically with kernel size k) to expand spatial features. Given the input feature map $X\in {\mathbb{R}}^{C\times H\times W}$, where C, H, and W denote the channel number, height, and width, respectively, its output can be formulated as shown in Equation (1):

(1) $$Y_{\mathrm{SPPF}}=\Phi ({\left\{X_{i}\right\}}_{i=0}^{3}),\;X_{0}=X,X_{i}=P_{k}(X_{i-1}),i=1,2,3$$

where $P_{k}(\cdot )$ denotes a max-pooling operation with kernel size k, and Φ(⋅) represents channel-wise feature aggregation. Although this recursive structure enlarges the receptive field, all branches originate from the same-scale transformations, leading to high feature correlation and redundant information. Moreover, repeated pooling progressively smooths spatial details, which limits the representation capability for small-scale targets and fine-grained structures.

To address these limitations, the proposed MSPPD introduces a parallel multi-scale pooling architecture, which can be expressed as shown in Equation (2):

(2) $$Y_{MSPPD}=\Phi ({\left\{X,P_{k_{i}}(X)\right\}}_{i=1}^{3})$$

where $k_{i}\in \{k_{1},k_{2},k_{3}\}$ denote different pooling kernel sizes (e.g., 3, 5, and 7). Unlike the cascaded design in SPPF, this parallel structure enables multi-scale features to be directly extracted from the original input, thereby achieving true multi-scale receptive field modeling. As a result, it effectively reduces feature redundancy and alleviates detail loss caused by recursive pooling, leading to improved feature diversity and representation capability.

To further enhance local structural information, depthwise separable convolution is applied to refine the aggregated features. The mapping relationship is shown in Equation (3):

(3) $$F_{\mathrm{loc}}=F_{dw}(Y_{\mathrm{MSPPD}})$$

where $F_{dw}(\cdot )$ denotes depthwise separable convolution, which efficiently captures edge, texture, and fine-grained spatial details with low computational overhead.

To further control the receptive field at different spatial scales, dilation rates are carefully selected in the proposed MSPPD module. Specifically, a set of dilation rates {1,2,3} is adopted to construct a lightweight hierarchical multi-scale receptive field structure, aiming to balance local detail preservation and global context modeling without introducing excessive computational overhead.

Small dilation rates (r=1) capture fine-grained structures such as hotspots and weak thermal anomalies, while larger dilation rates (r=2,3) progressively enlarge the receptive field for shadow regions and occlusions. This design enables the network to maintain both high-resolution spatial sensitivity and sufficient global contextual awareness, improving robustness across objects of varying scales.

**Figure 4:**
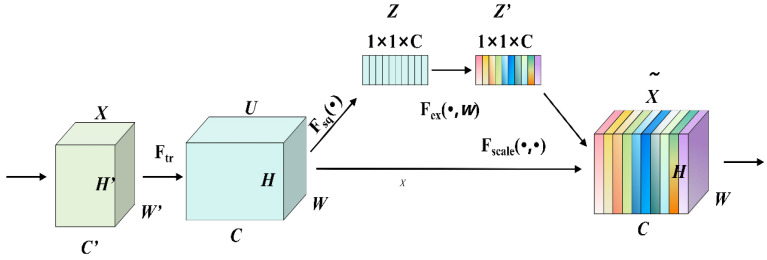
An illustration of the squeeze-and-excitation module.

Furthermore, to improve the discriminability of fused features, a squeeze-and-excitation (SE) attention mechanism is introduced for channel-wise adaptive recalibration [29], and its detailed structure is illustrated in Figure 4. A feature map of size C×W×H is obtained by compressing and encoding the spatial information on each channel into global features using global average pooling to acquire a feature map of size 1×1×C. Given $F_{\mathrm{loc}}\in {\mathbb{R}}^{C\times H\times W}$, the squeeze operation first encodes global spatial information into channel descriptors. The mapping relationship is shown in Equation (4):

(4) $$z_{c}=F_{sq}(F_{\mathrm{loc},c})=\frac{1}{HW}\sum_{i=1}^{H}\sum_{j=1}^{W}F_{\mathrm{loc},c}(i,j)$$

where $z_{c}$ denotes the global statistical feature of the *c*-th channel, and z represents the channel descriptor vector. $F_{sq}$ is the Squeeze operation definition, and i, j are pixel location variables.

In the excitation stage, channel dependencies are modeled via a two-layer fully connected transformation. The mapping relationship is described by Equation (5):

(5) $$s=F_{ex}(z,w)=\sigma (W_{2}\delta (W_{1}z))$$

where $F_{ex}$ is the Excitation operation definition, z is the Squeeze operation output, $W_{1}$ and $W_{2}$ are learnable parameters corresponding to channel reduction and restoration, respectively, δ(⋅) denotes the ReLU activation function, and σ(⋅) represents the sigmoid function. The resulting vector encodes channel-wise importance weights.

Finally, the reweighting operation produces the output feature. The mapping relationship is shown in Equation (6):

(6) $$Y_{c}=F_{scale}(F_{\mathrm{loc},c},s_{c})=s_{c}\cdot F_{\mathrm{loc},c}$$

where $F_{scale}$ is the definition of the Reweight operation, and $s_{c}$ is the output matrix channel of the Excitation operation.

Through the above formulation, MSPPD integrates multi-scale contextual modeling, local feature enhancement, and channel-wise adaptive recalibration into a unified framework. Compared with the conventional SPPF, which relies on recursive single-scale pooling, the proposed design shifts to parallel multi-branch modeling, resulting in reduced information redundancy, enhanced feature diversity, and improved representation capability. In practice, this leads to stronger adaptability to scale variations, more stable gradient propagation, and improved detection performance, particularly for small-scale targets and complex scenarios.

### 2.5. Gradient-Aware Fusion Module

In YOLO11, the original mid-to-late fusion typically adopts a simple concatenation (Concat) operation to merge visible and infrared features, which can be expressed in Equation (7):

(7) $$F_{\mathrm{fused}}=\mathrm{Concat}(F_{\mathrm{RGB}},F_{\mathrm{IR}})$$

Although this approach is straightforward and enables direct integration of multimodal information along the channel dimension, it essentially performs a linear stacking operation without distinguishing the relative importance of different modalities. As a result, it lacks the capability to effectively model cross-modal complementary information. In complex real-world scenarios, RGB images are susceptible to illumination variations and reflections, whereas infrared images primarily capture thermal information, leading to significant differences in semantic representation. Consequently, naive concatenation may introduce redundant information and even noise, thereby degrading subsequent feature extraction and detection performance.

**Figure 5:**
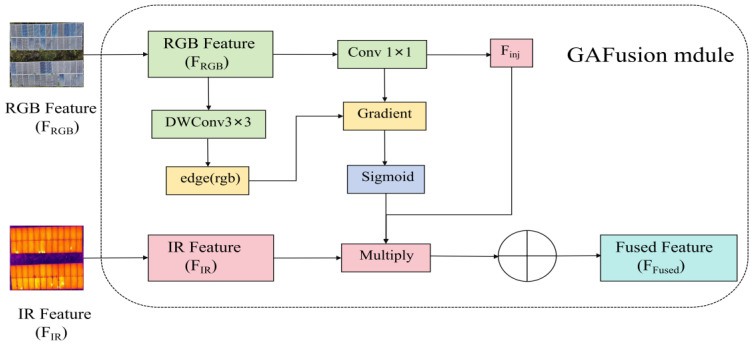
GAFusion module.

To address this issue, the proposed method replaces part of the Concat operations with the gradient-aware fusion (GAFusion) module, which introduces a gradient-guided mechanism for adaptive feature fusion. The architecture of the GAFusion module is illustrated in Figure 5. The fusion process first constructs spatial weights based on local variations in RGB features. By modeling the difference between the original features and their smoothed counterparts, a gradient response can be obtained from Equation (8):

(8) $$G=|F_{\mathrm{RGB}}-S(F_{\mathrm{RGB}})|,\;M=\sigma (G)$$

where S(⋅) denotes a depthwise convolution operator with fixed averaging initialization, serving as a local structural smoothing function, and σ(⋅) is the sigmoid activation function. The term G represents a high-frequency residual map, which captures local structural variations such as edges, textures, and boundary responses in visible-light features. The resulting mas M∈[0,1] is formulated as a spatial gating function, which adaptively measures the structural importance of visible features and determines their contribution during cross-modal fusion.

Subsequently, a 1×1 convolution is applied to the RGB features to perform channel mapping and compression, yielding the injected features, which can be expressed in Equation (9):

(9) $$F_{\mathrm{inj}}={\mathrm{Conv}}_{1\times 1}(F_{\mathrm{RGB}})$$

This transformation not only linearly projects RGB features into a space compatible with infrared features but also facilitates cross-modal channel alignment and semantic reconstruction, thereby enhancing the effectiveness of feature fusion.

Finally, the injected features are modulated by the spatial weights and fused with the infrared features through a weighted aggregation, producing the final output in Equation (10):

(10) $$F_{\mathrm{fused}}=F_{\mathrm{IR}}+M⊙F_{\mathrm{inj}}$$

In this formulation, the fusion process can be interpreted as a gradient-guided residual gating mechanism, where: $F_{\mathrm{IR}}$ acts as a thermal prior representation, providing robust thermal intensity information; and $M⊙F_{\mathrm{inj}}$ serves as a structure-aware residual correction term, selectively injecting visible structural cues into infrared features.

Therefore, the proposed GAFusion can be formally expressed in Equation (11):

(11) $$F_{\mathrm{fused}}=F_{\mathrm{IR}}+\phi (F_{RGB},S(F_{RGB}))$$

where ϕ(⋅) denotes a gradient-guided adaptive fusion function, which jointly models structural saliency and modality alignment.

Compared with the conventional Concat operation, GAFusion implements a nonlinear feature fusion mechanism through spatial saliency weighting and channel mapping. This allows RGB features to selectively enhance infrared features only in discriminative regions, effectively suppressing redundant information and noise. Meanwhile, the proposed method maintains feature dimensional consistency, avoiding the additional computational burden caused by channel expansion. As a result, it improves fusion quality while preserving computational efficiency.

Furthermore, since gradient information is inherently sensitive to edges and structural variations, the proposed module demonstrates stronger robustness and discriminative capability in tasks involving small object detection, occlusion recognition, and fine-grained defect identification, such as hotspots. Overall, GAFusion transforms the fusion paradigm from linear concatenation to spatially adaptive weighted fusion at the formulation level, shifting multimodal integration from passive aggregation to active selection and enhancement. This significantly improves the complementary utilization of visible and infrared information, thereby enhancing the detection performance of YOLO11 in complex environments.

### 2.6. Dynamic Convolution and Element-Wise Scaling Detection Head

In YOLO11, the original detection head (Detect) mainly consists of a convolutional regression branch and a convolutional classification branch. The regression branch directly outputs bounding box offsets through consecutive convolutional layers, while the classification branch predicts category probabilities via convolution. The overall formulation can be expressed in Equation (12):

(12) $$O_{\mathrm{orig}}=\mathrm{Concat}({\mathrm{Conv}}_{\mathrm{reg}}(F),{\mathrm{Conv}}_{\mathrm{cls}}(F))$$

Although this structure is simple and computationally efficient, its use of fixed convolution kernels limits the ability to adaptively adjust feature extraction according to input content. Consequently, it struggles to effectively model variations in object scale, shape, and background complexity in real-world scenarios. This limitation is particularly evident in small object detection and heavily occluded scenes, where localization errors and misclassification are more likely to occur.

**Figure 6:**
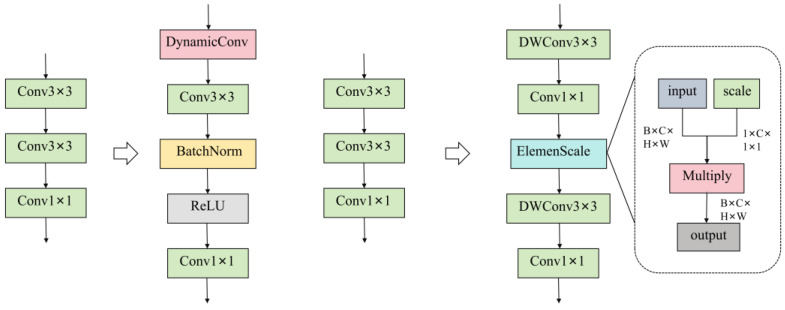
Comparison of regression and classification branches in Detect before and after improvement.

**Figure 7:**
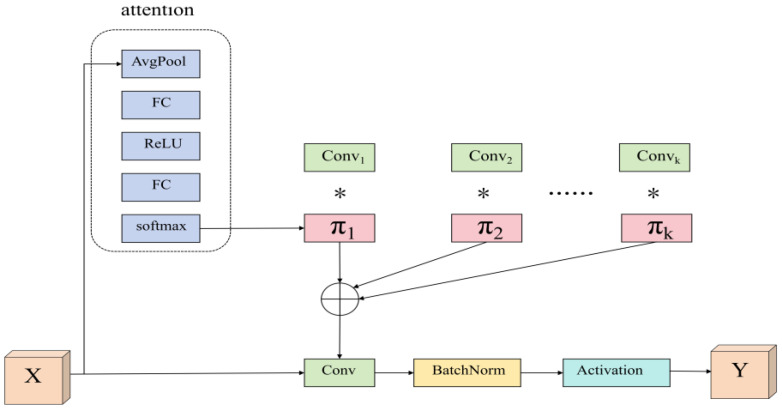
Dynamic convolution module.

To address these issues, the original Detect head is replaced with the proposed Detect-DEhead (as shown in Figure 6). The design is inspired by the dynamic head paradigm and further enhanced by incorporating dynamic modeling strategies from [30] and the dynamic convolution (DynamicConv) module proposed by Tong et al. [31] (as illustrated in Figure 7). Specifically, an input-adaptive mechanism is introduced into the detection head.

In the regression branch, traditional convolution is replaced by DynamicConv. Dynamic convolution generates input-dependent convolution kernels conditioned on the input feature map. Let F denote the input feature, and the dynamic convolution can be formulated as shown in Equations (13) and (14):

(13) $$W_{dyn}=G(F)$$

(14) $$F^{\prime }=\sigma (BN(W_{dyn}*F))$$

where G(⋅) represents a kernel generation function conditioned on global feature statistics, $W_{dyn}$ denotes the dynamically generated convolution kernel, and BN(⋅) and σ(⋅) denote batch normalization and ReLU activation, respectively. This module generates input-dependent convolutional kernel weights, enabling spatially adaptive feature extraction. As a result, the model can learn differentiated response patterns for different object regions. In addition, batch normalization (BatchNorm) and ReLU activation are applied after dynamic convolution to stabilize feature distributions and enhance nonlinear representation capabilities, thereby improving the accuracy and robustness of bounding box regression. The mapping relationship is shown in Equation (15):

(15) $$F_{\mathrm{reg}}=\mathrm{ReLU}(\mathrm{BN}(\mathrm{Dynamic}\mathrm{Conv}(F)))$$

In the classification branch, to reduce computational complexity while improving feature representation efficiency, a combination of depthwise separable convolution (DWConv) and pointwise convolution is adopted. Meanwhile, a lightweight channel modulation module, namely ElementScale, performs channel-wise adaptive reweighting. Given an input feature $F\in {\mathbb{R}}^{C\times H\times W}$, the scaling operation is defined as shown in Equations (16) and (17):

(16) α=σ(GAP(F))

(17) $$F_{c^{\prime }}=\alpha_{c}\cdot F_{c}$$

where GAP(⋅) denotes global average pooling, α is the learned channel importance vector, and each channel is rescaled adaptively according to its semantic contribution.

ElementScale is introduced to adaptively rescale feature channels, thereby enhancing the response to discriminative semantic information. The classification branch can be expressed in Equation (18):

(18) $$F_{cls}=DW\mathrm{Conv}(F)\cdot s$$

where the channel scaling coefficient s is learned by the ElementScale module and can be expressed as Equation (19):

(19) s=ElementScale(F)

This mechanism dynamically adjusts feature responses according to the importance of each channel, enabling more flexible and efficient multi-class discrimination.

Finally, the outputs of the regression and classification branches are concatenated to produce the final output of the detection head, which is given in Equation (20):

(20) $$O_{\mathrm{DEhead}}=\mathrm{Concat}(F_{\mathrm{reg}},F_{\mathrm{cls}})$$

Compared with the original Detect head, Detect-DEhead offers several advantages. First, by introducing input-dependent weight generation through DynamicConv, the regression branch achieves spatially adaptive modeling, significantly improving bounding box localization accuracy. Second, the ElementScale module enables fine-grained channel-wise modulation in the classification branch, allowing the model to focus on more discriminative features and thus improving classification performance. Third, the incorporation of DWConv effectively reduces computational cost, enabling the improved detection head to achieve better performance while maintaining low parameter count and GFLOPs.

This design transforms the detection head from fixed convolutional modeling to dynamic adaptive modeling. It demonstrates stronger robustness and generalization capability in complex backgrounds, multi-scale object scenarios, and occluded conditions, thereby significantly enhancing the detection accuracy and stability of YOLO11 in practical applications.

### 2.7. Shape-IoU

To further evaluate localization accuracy, intersection over union (IoU)-related metrics are introduced to analyze the overlap between predicted bounding boxes and ground truth boxes. In object detection tasks, bounding box regression is typically optimized based on IoU and its variants. IoU measures the degree of overlap between the predicted box and the ground truth box, which can be defined as Equation (21):

(21) $$IoU=\frac{\left|B\cap B_{gt}\right|}{\left|B\cup B_{gt}\right|}$$

where the predicted box and ground truth box are denoted as B and $B_{\mathrm{gt}}$, respectively. However, when there is no overlap between the two boxes, IoU becomes zero and fails to provide effective gradients, thereby hindering model convergence.

To address this issue, YOLO11 adopts the complete IoU (CIoU) loss function for bounding box regression, which can be expressed as Equation (22):

(22) $$CIoU=IoU-\frac{\rho^{2}(b,b_{gt})}{c^{2}}-\alpha v$$

where $b=(x_{c},y_{c})$ and $b_{gt}=(x_{c}^{gt},y_{c}^{gt})$ represent the center points of the predicted box and the ground truth box, respectively, and ρ(⋅) denotes the Euclidean distance and corresponds to the diagonal length of the minimum enclosing box. The aspect ratio consistency term is defined as Equations (23) and (24):

(23) $$v=\frac{4}{\pi^{2}}{\left(\arctan\frac{w_{gt}}{h_{gt}}-\arctan\frac{w}{h}\right)}^{2}$$

(24) $$\alpha =\frac{v}{(1-IoU)+v}$$

Although CIoU improves regression accuracy by incorporating center distance and aspect ratio constraints, it mainly focuses on the relative geometric relationship between the predicted box and the ground truth box, neglecting the influence of the object’s inherent shape and scale characteristics on the regression results. Studies have shown that, under the same offset conditions, objects with different shapes or scales may produce different IoU variations, and this discrepancy is particularly pronounced for elongated objects and small-scale targets. Therefore, traditional IoU-based loss functions still exhibit limitations in complex scenarios.

To overcome these limitations, the Shape-IoU loss is introduced in this work to replace CIoU for bounding box regression. This method further models the shape and scale information of objects on the basis of IoU. By incorporating a direction-aware weighting mechanism, different directional offset errors are assigned different levels of importance [23]. Specifically, the horizontal and vertical weighting coefficients are first defined as in Equations (25) and (26):

(25) $$w_{w}=\frac{2{\left(w_{gt}\right)}^{scale}}{{\left(w_{gt}\right)}^{scale}+{\left(h_{gt}\right)}^{scale}}$$

(26) $$w_{h}=\frac{2{\left(h_{gt}\right)}^{scale}}{{\left(w_{gt}\right)}^{scale}+{\left(h_{gt}\right)}^{scale}}$$

Based on this, a shape-aware center distance term is constructed as in Equation (27):

(27) $$distance_{shape}=w_{h}\cdot \frac{{\left(x_{c}-x_{g}^{c}\right)}^{2}}{c^{2}}+w_{w}\cdot \frac{{\left(y_{c}-y_{g}^{c}\right)}^{2}}{c^{2}}$$

Meanwhile, a shape consistency constraint is introduced to measure the discrepancy between the predicted box and the ground truth box in terms of width and height:

(28) $$\Omega_{shape}=\sum_{t\in \{w,h\}}{\left(1-e^{-\omega_{t}}\right)}^{\theta },\theta =4$$

where:

(29) $$\omega_{w}=w_{h}\cdot \frac{\left|w-w_{gt}\right|}{\max(w,w_{gt})}$$

(30) $$\omega_{h}=w_{w}\cdot \frac{\left|h-h_{gt}\right|}{\max(h,h_{gt})}$$

Finally, the Shape-IoU loss function is defined as in Equation (31):

(31) $$L_{shape-IoU}=1-IoU+distance_{shape}+0.5\cdot \Omega_{shape}$$

Compared with CIoU, Shape-IoU incorporates direction-sensitive and scale-adaptive mechanisms, enabling a more refined characterization of object geometry. In PV module fault detection tasks, defects such as occlusions and shadows often exhibit irregular shapes and small-scale characteristics. Traditional methods struggle to accurately model their spatial distributions, whereas Shape-IoU can strengthen constraints along the shorter edges, thereby improving localization accuracy and reducing false detections.

## 3. Results

### 3.1. Experimental Environment and Parameter Settings

All model training and evaluation experiments in this study were conducted on a high-performance workstation. The experimental platform is equipped with an NVIDIA GeForce RTX 4090 GPU (24 GB memory) and runs on the Ubuntu 20.04 operating system. The deep learning framework is based on Ultralytics YOLO11 (version 8.3.75), built upon PyTorch 2.2.1, with the runtime environment configured using Python 3.11.14. CUDA 12.1 is enabled to accelerate both training and inference processes.

During training, the input image size is uniformly set to 640 × 640. The model is trained for 200 epochs with a batch size of 32, and the stochastic gradient descent (SGD) optimizer is used for parameter updates. All experiments are conducted using a single GPU (device = 0), with the number of data loading workers set to four.

For the dual-modality visible–infrared fusion task, the model input is configured as a six-channel representation, enabling joint modeling of RGB and infrared information through a customized dual-modality input strategy. In addition, to ensure experimental fairness and reduce the impact of data augmentation, color-related augmentation operations are disabled during training (HSV-H, HSV-S, and HSV-V are all set to 0). To guarantee the fairness and reliability of comparative evaluations, all experiments were conducted under identical training settings, including the same input resolution, optimizer configuration, training epochs, batch size, and data augmentation strategy. Furthermore, all models were trained and evaluated on the same dataset splits and hardware environment to minimize the influence of external factors on performance evaluation.

This experimental configuration ensures stable training while fully leveraging the computational advantages of high-performance GPUs for dual-modality object detection, providing a reliable foundation for subsequent performance comparisons and ablation studies.

### 3.2. Model Evaluation Metrics

To comprehensively evaluate the detection performance and inference efficiency of the proposed model in PV small-object fault detection tasks, precision, recall, and mean average precision (mAP) are adopted as the primary evaluation metrics. The corresponding formulations are defined as follows.

Recall measures the model’s ability to detect true objects and is defined as the ratio of correctly detected positive samples to all ground truth positive samples. Precision reflects the accuracy of the model’s predictions and is defined as the proportion of correctly predicted positive samples among all predicted positive samples:

(32) $$Recall=\frac{TP}{TP+FN}\times 100\%$$

(33) $$Precision=\frac{TP}{TP+FP}\times 100\%$$

where TP (true positive) denotes the number of correctly detected target samples, FP (false positive) represents the number of samples incorrectly predicted as targets, and FN (false negative) denotes the number of ground truth targets missed by the model.

In object detection tasks, average precision (AP) is used to evaluate the detection performance across different recall levels. It is defined as the area under the precision–recall (P–R) curve:

(34) $$AP=\int_{0}^{1}P(R)dR$$

Furthermore, to eliminate bias introduced by single-class evaluation, mean average precision (mAP) is employed as the overall performance metric, which is defined as the average of AP values over all categories:

(35) $$mAP=\frac{1}{K}\sum_{i=1}^{K}AP_{i}\times 100\%$$

where K denotes the total number of object categories and $AP_{i}$ represents the average precision of the *i*-th category.

In addition to accuracy-related metrics, to comprehensively assess the deployability and computational efficiency of the model in practical applications, several efficiency indicators are also introduced, including the number of parameters (Parameters), model size (Model Size), floating-point operations (GFLOPs), and inference speed measured in frames per second (FPS). Specifically, Parameters and GFLOPs are used to evaluate model complexity and computational cost, while FPS reflects the real-time inference capability on GPU platforms.

Through these multi-dimensional evaluation metrics, different models can be objectively and comprehensively compared from both accuracy and efficiency perspectives.

### 3.3. Feature-Level Fusion Strategy Analysis

**Figure 8:**
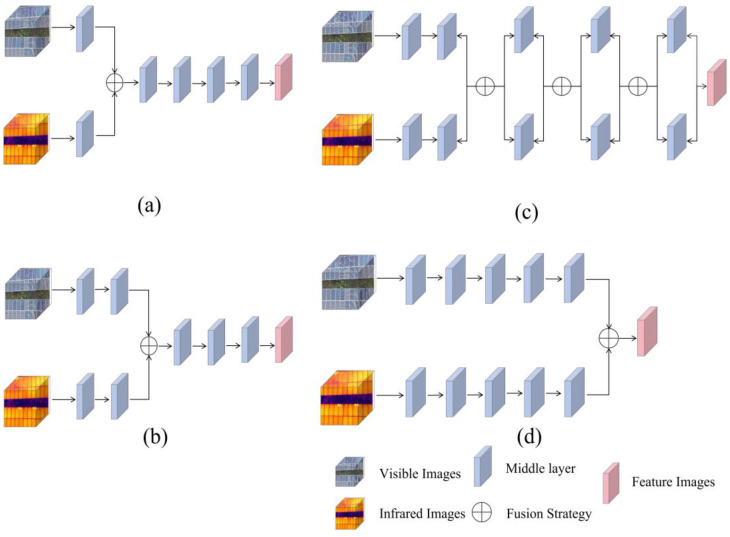
(**a**) Early fusion; (**b**) mid fusion; (**c**) mid-to-late fusion; (**d**) late fusion.

In multimodal object detection tasks, feature-level fusion serves as a critical bridge between raw data representation and high-level semantic understanding. According to the stage at which fusion occurs within the network, feature-level fusion strategies are typically categorized into early fusion, mid fusion, mid-to-late fusion, and late fusion. Different fusion stages exhibit distinct characteristics in terms of information retention, semantic representation capability, and computational complexity. The specific structures of different fusion strategies are illustrated in Figure 8.

To evaluate the impact of different feature-level fusion stages on dual-modality PV fault detection performance, comparative experiments were conducted under the same network framework using early fusion, mid fusion, mid-to-late fusion, and late fusion. The quantitative results are presented in Table 1, where the evaluation metrics include precision (P), recall (R), mAP@0.5, number of parameters (Params), computational cost (GFLOPs), and model size (Model Size).

From the perspective of detection performance, mid-to-late fusion achieves the best overall results, showing superior accuracy and recall among all strategies. This indicates a stronger capability in reducing missed detections and improving detection completeness, which is particularly beneficial for small-scale and weak-feature faults such as hotspots and diode short circuits. Although late fusion attains slightly higher precision, its lower recall limits its overall effectiveness. In contrast, mid-to-late fusion provides a better balance between precision and recall, resulting in more stable performance.

In terms of model complexity, mid-to-late fusion achieves a favorable trade-off between performance and computational cost. Compared with early fusion, it introduces a moderate increase in complexity, while remaining more efficient than late fusion in both parameter scale and computational burden. Additionally, its relatively smaller model size demonstrates better suitability for practical deployment.

Overall, different fusion strategies exhibit inherent limitations in multimodal detection tasks. Early fusion directly combines heterogeneous features at shallow layers, making it sensitive to noise and prone to degraded feature representation. Mid fusion partially alleviates modality discrepancies but remains limited in high-level semantic modeling. Late fusion improves precision through decision-level aggregation but lacks deep feature interaction and incurs higher computational costs.

In contrast, mid-to-late fusion enables more effective cross-modal interaction at higher semantic levels, achieving a better balance between detection performance and efficiency. However, most existing methods rely on static fusion strategies and lack adaptive modeling of modality discrepancies. Therefore, incorporating modality-aware mechanisms and adaptive fusion strategies remains essential for further improving multimodal feature representation.

### 3.4. Single-Modality and Dual-Modality Analysis

As shown in Table 2, the comparison results indicate that the dual-modality model incorporating both visible and infrared information achieves superior overall performance in PV module fault detection, particularly for easily confused fault types such as occlusions and shadows. Specifically, the average precision (AP) for occlusions increases from 78.2% to 85.5%, while that for shadows improves from 77.2% to 80.2%, demonstrating that multimodal fusion effectively compensates for the limitations of single-modality perception. The underlying reason for this performance gain lies in the complementary characteristics of the two modalities. Infrared images are sensitive to temperature variations and can effectively highlight thermal anomalies such as hotspots and electrical faults, whereas visible images provide rich structural and textural information. In occlusion and shadow scenarios, a single infrared modality often struggles to distinguish true thermal anomalies from pseudo-anomalies caused by occlusion or illumination variations, leading to false detections or missed detections. By contrast, dual-modality fusion leverages the structural and semantic cues from visible images to constrain infrared features, thereby reducing interference caused by environmental factors such as illumination changes and partial occlusions. Furthermore, the dual-modality model improves mAP@0.5 from 84.7% to 86.7%, further confirming its advantage in overall detection accuracy. This indicates that feature fusion not only enhances discrimination capability in complex scenarios (e.g., occlusion and shadow) but also improves model generalization and robustness. Therefore, for PV module fault detection, dual-modality methods provide a more effective and practical solution by reducing false detections and improving the ability to distinguish fault types under complex conditions.

### 3.5. Ablation Experiment

**Figure 9:**
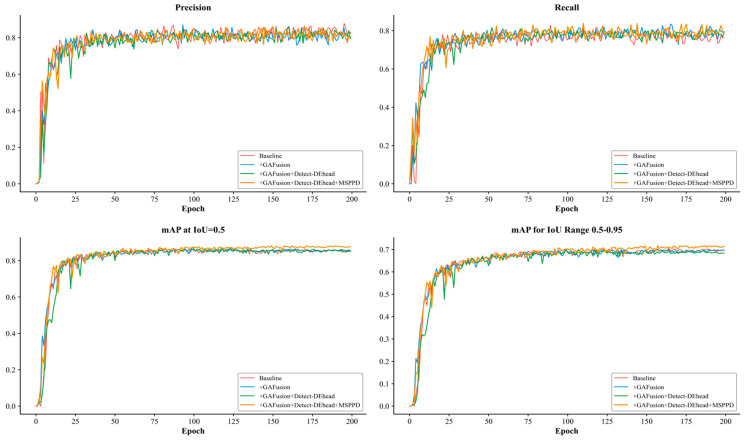
Comparison of performance curves between the baseline and incrementally modified models.

To evaluate the contribution of each proposed component, a series of ablation experiments were conducted on the YOLO11-based dual-modality detection framework. Specifically, three modules—gradient-aware fusion (GAFusion), multi-scale pyramid pooling and dilated convolution (MSPPD), and the dynamic convolution and element-wise scaling detection head (Detect-DEhead)—were progressively introduced. Among them, GAFusion replaces part of the Concat operations in the feature fusion stage, MSPPD replaces the SPPF module in the backbone, and Detect-DEhead substitutes the original Detect head. The experimental results are summarized in Table 3 and Figure 9, where Test 1 denotes the baseline model.

When only the GAFusion module is introduced (Test 2), the recall increases from 81.2% to 82.0% (+0.8%), and mAP@0.5 improves from 86.7% to 87.0% (+0.3%). Meanwhile, the computational cost is reduced from 11.42 GFLOPs to 10.45 GFLOPs (−8.49%). These results indicate that GAFusion enhances cross-modal feature fusion while reducing computational complexity, thereby improving the model’s ability to detect target instances. When only the MSPPD module is applied (Test 3), the number of parameters decreases from 4.3 M to 4.0 M (−6.98%), and the model size is reduced from 9.1 MB to 8.5 MB (−6.59%), while maintaining a comparable mAP@0.5 of 86.9%. This demonstrates that MSPPD effectively enhances multi-scale feature representation while maintaining a lightweight design. When replacing the detection head with Detect-DEhead (Test 4), the precision improves from 80.4% to 83.0% (+2.6%), and mAP@0.5 increases to 87.1% (+0.4%). This indicates that the improved detection head enhances both classification and localization capabilities, thereby improving overall detection accuracy.

Furthermore, when combining two modules, the model exhibits clear synergistic improvements. For instance, when both GAFusion and MSPPD are introduced (Test 5), the number of parameters is reduced to 3.9 M (−9.30% compared to the baseline), and GFLOPs decrease to 10.19 (−10.77%), indicating strong complementarity between feature extraction and feature fusion modules. When all three modules are jointly applied (Test 8), the model achieves the best overall performance. Specifically, mAP@0.5 increases to 88.1%, representing a gain of 1.4% over the baseline. Meanwhile, the number of parameters is reduced to 3.7 M (−13.95%), GFLOPs decrease to 9.37 (−17.95%), and the model size is reduced from 9.1 MB to 7.9 MB (−13.19%). These results demonstrate that the three modules form an effective synergy across feature extraction, cross-modal fusion, and detection head optimization, significantly improving detection accuracy while reducing computational complexity.

Although the introduction of these modules leads to a decrease in inference speed (FPS) from 71.85 to 59.55, the reduction remains within an acceptable range for real-time photovoltaic inspection applications. Meanwhile, the proposed method achieves higher detection accuracy with reduced computational complexity and model size, demonstrating a favorable trade-off between detection performance and deployment efficiency. Therefore, the proposed framework remains suitable for resource-constrained drone-based PV inspection scenarios.

In addition, Shape-IoU is introduced in Table 4. Although a slight decrease in precision is observed, both mAP and recall are significantly improved, indicating enhanced localization performance. Specifically, recall increases from 78.5% to 84.9%, demonstrating that Shape-IoU provides more stable regression for difficult defect instances and effectively reduces missed detections.

This improvement is particularly beneficial for photovoltaic defects with elongated or irregular geometries, such as shadows and occlusions. Compared with conventional IoU-based losses, Shape-IoU introduces direction-aware weighting and shape consistency constraints, enabling more accurate boundary alignment and better sensitivity to aspect-ratio variations. As a result, the proposed method achieves more robust localization performance in complex scenarios without modifying the network architecture or introducing additional computational overhead.

The proposed GAFusion, MSPPD, Detect-DEhead modules, and the Shape-IoU loss all contribute positively to model performance. Their combined application further improves detection accuracy while reducing model complexity, thereby validating the effectiveness of the proposed method.

### 3.6. Comparative Experiments

#### 3.6.1. Comparison with Different Models

**Figure 10:**
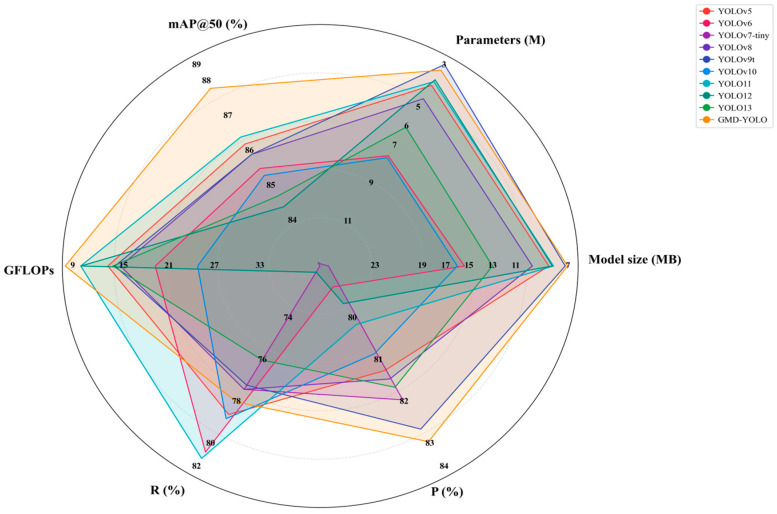
Radar chart comparison of different models.

Table 5 and Figure 10 present a comparative analysis of detection performance and computational complexity for different YOLO versions (unless otherwise specified, the nano (*n*) variant is used throughout this paper), ranging from YOLOv5 to YOLOv13, under a unified mid-to-late fusion framework. It can be observed that different YOLO variants exhibit notable differences in detection accuracy and computational efficiency.

From the perspective of detection performance, YOLO11 achieves the best overall results, particularly in terms of recall and overall detection accuracy. Specifically, YOLO11 attains a recall of 81.2%, which is the highest among all compared models, indicating its superior ability to reduce missed detections under dual-modality fusion conditions. This advantage is particularly beneficial for detecting small-scale or weak-feature targets, such as hotspots and diode short circuits. In addition, YOLO11 achieves an mAP@0.5 of 86.7%, which is among the highest across all models, outperforming YOLOv5, YOLOv6, YOLOv7-tiny, and YOLOv12, while remaining competitive with YOLOv8 and YOLOv9. In terms of model complexity, YOLO11 also demonstrates a favorable balance between efficiency and performance. Its parameter count is 4.3 M, significantly lower than that of YOLOv7-tiny (13.9 M) and YOLOv10 (8.3 M) and comparable to YOLOv8 (5.2 M) and YOLOv12 (4.2 M). Regarding computational cost, YOLO11 requires 11.42 GFLOPs, which is relatively low among all models, only slightly higher than YOLOv12 but notably lower than YOLOv5, YOLOv7-tiny, and YOLOv10. Meanwhile, the model size of YOLO11 is only 9.1 MB, effectively reducing storage and deployment costs while maintaining high detection performance.

A comprehensive comparison shows that some earlier models (e.g., YOLOv5) have advantages in terms of parameter count and model size but exhibit inferior detection performance. In contrast, more recent models (e.g., YOLOv7-tiny and YOLOv10) achieve improvements in certain metrics at the expense of significantly increased computational complexity. Compared with these models, YOLO11 achieves an optimal trade-off between detection accuracy and computational efficiency under the dual-modality mid-to-late fusion framework, characterized by high recall, high mAP@0.5, and relatively low computational costs. These results demonstrate that YOLO11 is well-suited to the mid-to-late fusion architecture and further justify its selection as the baseline model for dual-modality PV fault detection.

**Figure 11:**
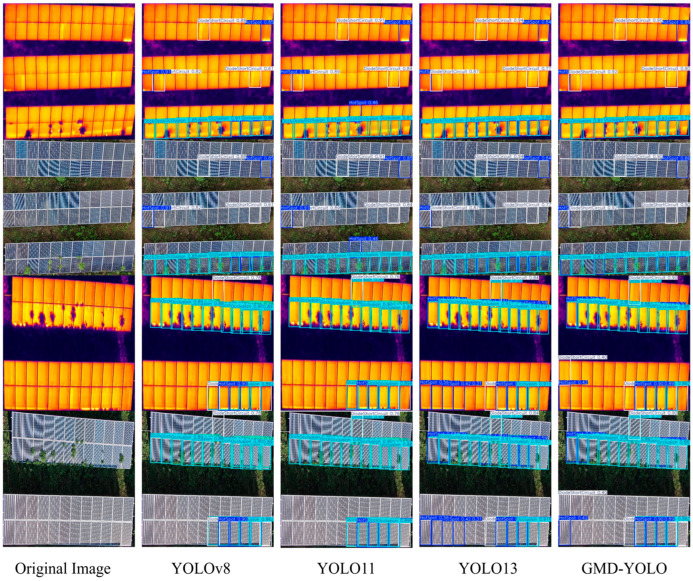
Visual comparison of detection results across different models.

To provide a more intuitive evaluation of detection performance in dual-modality scenarios, a set of representative visible and infrared image pairs is selected, and the detection results of YOLOv8, YOLO11, YOLOv13, and the proposed method are compared. As shown in Figure 11, different models exhibit varying capabilities in object localization accuracy and adaptation to complex scenes.

From the visualization results in Figure 11, YOLOv8 can perform basic detection of large PV modules; however, it tends to miss targets or produce inaccurate localization in local regions, especially where thermal features are weak or background textures are complex. This indicates that its utilization of multimodal information still relies heavily on single-modality feature responses. YOLO11 improves detection completeness, with most components being correctly detected. Nevertheless, some bounding box misalignment and duplicate detections can still be observed, particularly in densely arranged or occluded scenarios. This suggests that although feature fusion is enhanced, the complementary information between modalities is not fully exploited. YOLOv13 further improves detection continuity, producing more stable bounding results and reducing missed detections. However, in small-scale or weak-response regions, the predicted bounding boxes are still not sufficiently well-aligned, indicating limited sensitivity to subtle anomalies.

In contrast, the proposed method demonstrates more robust and stable performance across these scenarios. On the one hand, object boundaries are more accurately localized, with bounding boxes better aligned with the actual positions of PV modules. On the other hand, the method maintains strong detection completeness even in regions with weak thermal anomalies or partial occlusions. The number of false detections and redundant bounding boxes is significantly reduced, indicating that the model can effectively integrate visible and infrared information to generate more discriminative feature representations.

The improved model exhibits clear advantages in multimodal feature utilization and adaptation to complex environments, which is consistent with the trends observed in the quantitative evaluation results.

#### 3.6.2. Illumination Robustness Experiments

In practical drone-based photovoltaic inspection scenarios, environmental factors such as low-light conditions, overcast weather, and uneven lighting conditions can significantly degrade image quality and affect fault detection performance. In particular, low-light conditions may weaken structural texture information and increase sensor noise, while overcast weather often reduces image contrast and blurs fine-grained details. These factors increase the difficulty of multimodal feature extraction, feature alignment, and cross-modal fusion under complex outdoor environments.

To evaluate the robustness and adaptability of the proposed framework under adverse illumination conditions, additional illumination degradation experiments were conducted on both visible-light and infrared datasets. Specifically, low-light conditions were simulated through brightness attenuation and Gaussian noise injection, whereas overcast weather was simulated using contrast suppression, grayscale blending, and image smoothing operations. Through these simulated environmental disturbances, the stability and environmental adaptability of the proposed multimodal fusion strategy can be further analyzed under complex imaging conditions commonly encountered in UAV-based PV inspection tasks.

The quantitative comparison results are presented in Table 6. Under overcast weather conditions, the proposed GMD-YOLO achieves an mAP50 of 85.4%, outperforming the baseline YOLO11 model. Under low-light conditions, GMD-YOLO further improves precision from 79.4% to 81.0% and increases the mAP50 from 85.5% to 85.7%, demonstrating stronger robustness under degraded illumination environments. Although the proposed method introduces a reduction in FPS due to the enhanced multimodal feature modeling process, it still maintains acceptable real-time inference capability for practical UAV-based PV inspection tasks.

**Figure 12:**
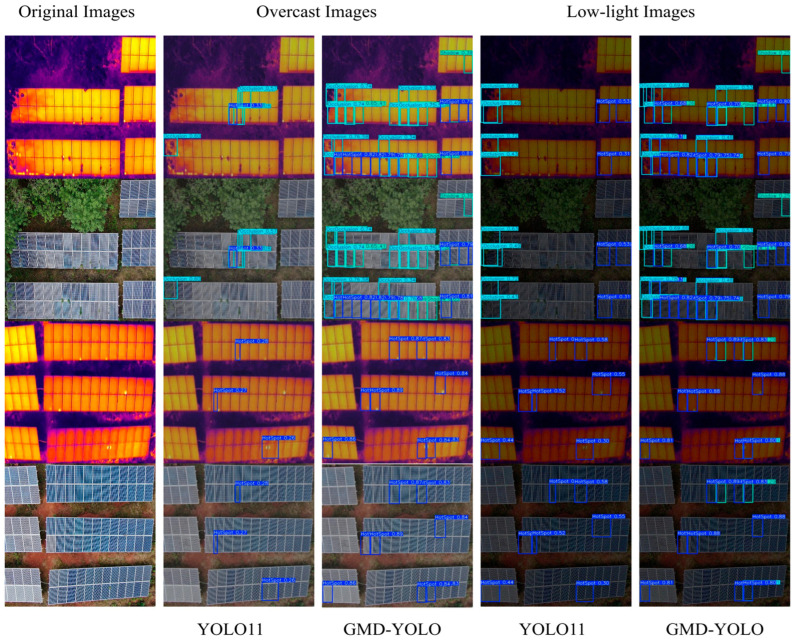
Visual comparison between the baseline and proposed models under different illumination conditions.

Furthermore, the visualization results shown in Figure 12 demonstrate that the proposed GMD-YOLO exhibits significantly improved detection stability and localization capabilities under both overcast weather and low-light conditions. Compared with the original YOLO11 model, the proposed method effectively reduces missed detections and improves localization accuracy for weak-contrast and small-scale faults. In particular, the proposed framework maintains clearer feature responses and more accurate fault localization in challenging regions with degraded texture details and weakened thermal contrast, further validating the effectiveness of the proposed multimodal fusion strategy under complex environmental conditions.

#### 3.6.3. Comparison on Different Datasets

To further validate the effectiveness and generalization capability of the proposed method, comparative experiments are conducted on the publicly available FLIR Thermal dataset. The improved GMD-YOLO model is compared with the original YOLO11 dual-modality model, and the results are presented in Table 7.

As shown in the results, the proposed GMD-YOLO outperforms the baseline YOLO11 across multiple evaluation metrics. Specifically, precision increases from 78.2% to 80.4% (+2.2 percentage points), recall improves from 61.9% to 63.5% (+1.6 percentage points), and mAP@0.5 rises from 69.8% to 72.5% (+2.7 percentage points). These results demonstrate that the introduction of the GAFusion, MSPPDD, and Detect-DEhead modules significantly enhances the model’s object detection capability in complex scenarios. Meanwhile, the improved model not only achieves better detection performance but also effectively reduces model complexity. Specifically, the number of parameters decreases from 4.3 M to 3.7 M (−13.95%), GFLOPs are reduced from 11.42 to 9.36 (−18.04%), and the model size is reduced from 9.1 MB to 7.9 MB (−13.19%). This indicates that the proposed method improves detection accuracy while simultaneously enhancing computational efficiency, resulting in a more lightweight model.

Furthermore, the experimental results on the public dataset demonstrate that the proposed method not only performs well on the self-collected PV dataset but also achieves consistent performance improvements across different scenarios. This confirms that the proposed modules exhibit strong cross-dataset generalization capability and can effectively enhance feature representation and detection robustness in complex infrared environments, thereby validating the practical applicability of the proposed approach.

**Figure 13:**
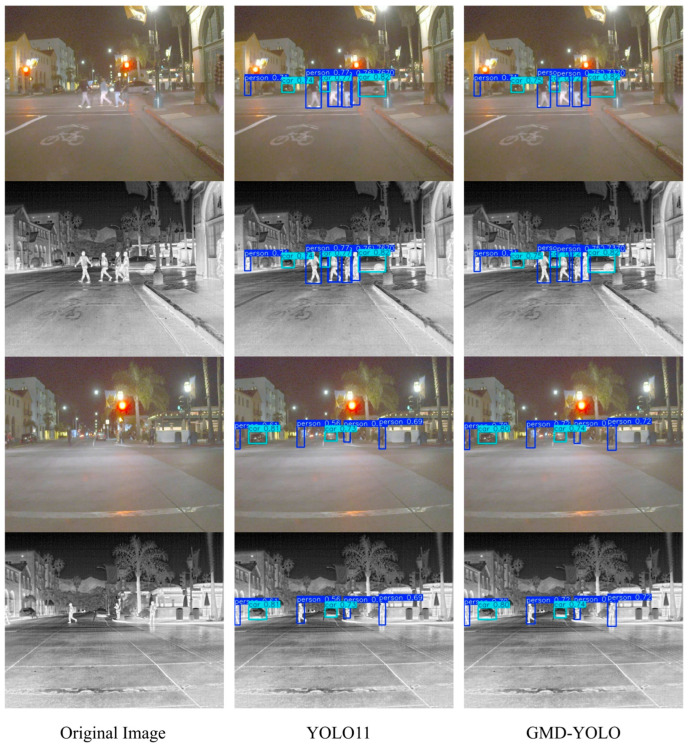
Visual comparison of detection results between the baseline and improved models on the FLIR Thermal dataset.

As shown in Figure 13, the original images, as well as the detection results of YOLO11 and the improved model, are presented for comparison.

Overall, YOLO11 demonstrates a certain level of capability in multimodal object detection and is able to identify the main pedestrian targets in the scene. However, its detection performance remains somewhat unstable under challenging conditions, such as complex illumination and low contrast. For example, in nighttime visible-light images, due to insufficient illumination and noise interference, some pedestrian targets exhibit low confidence scores or inaccurate localization. In infrared images, although the target contours are relatively clear, missed detections or incomplete bounding boxes still occur in regions with long distances or weak thermal responses.

In contrast, the improved model exhibits more stable detection performance across different modalities. On the one hand, in visible-light scenarios, the model can better suppress background noise and enhance the response to weak targets. On the other hand, in infrared images, it achieves more accurate recognition of distant or low-contrast targets, with improved completeness and alignment of bounding boxes. Moreover, it can be observed that the improved model produces more consistent detection results in multi-object scenarios, with fewer false positives and redundant detections. Cross-modality comparisons further reveal that the improved model is able to more effectively fuse visible and infrared information, resulting in more consistent detection outcomes across different modalities and demonstrating enhanced robustness. This indicates that the proposed improvements not only strengthen feature representation but also improve the model’s adaptability to complex environmental variations.

The visualization results demonstrate that the improved model outperforms the original YOLO11 in terms of detection stability, localization accuracy, and weak target recognition capability. This trend is consistent with the subsequent quantitative experimental results, further validating the effectiveness of the proposed method.

## 4. Discussion

The experimental results demonstrate that the proposed GMD-YOLO achieves consistent improvements in detection accuracy while reducing model complexity, indicating an effective balance between performance and efficiency. Compared with the baseline YOLO11 dual-modality framework, the gains in mAP@0.5, recall, and cross-dataset generalization suggest that the proposed modifications successfully mitigate key challenges in photovoltaic fault detection, particularly under complex environmental conditions.

From a feature representation perspective, the MSPPD module enhances multi-scale contextual modeling by replacing conventional cascaded pooling with a parallel multi-branch structure. This design effectively alleviates feature redundancy and detail loss, which are critical issues for small-scale and low-contrast defects such as hotspots and shadows. Meanwhile, the GAFusion module introduces a gradient-aware mechanism that enables spatially adaptive feature selection, allowing the model to focus on structurally informative regions (e.g., edges and textures) in visible images while suppressing irrelevant noise. In practical PV inspection scenarios, visible images may contain visually salient disturbances, such as reflections, illumination artifacts, and background textures, which do not correspond to actual thermal anomalies in infrared images. The proposed fusion strategy alleviates this complementary redundancy issue by adaptively emphasizing cross-modally consistent features while reducing the influence of thermally irrelevant visual responses. This significantly improves the quality of cross-modal feature interaction compared with traditional concatenation-based fusion strategies. Furthermore, the proposed Detect-DEhead improves both classification and localization performance through dynamic convolution and channel-wise scaling. The experimental results show that this module contributes notably to precision improvement, indicating its effectiveness in reducing false positives. In addition, the introduction of Shape-IoU further enhances localization accuracy and recall, particularly for irregular and small objects, which are common in PV fault scenarios.

Despite these advantages, several limitations remain. First, although the proposed fusion strategy improves cross-modal interaction, spatial misalignment between visible and infrared images remains a persistent challenge that may degrade performance under extreme conditions, such as large viewpoint shifts or severe thermal noise. Second, while the model achieves a reduction in parameters and GFLOPs, inference speed (FPS) experiences a trade-off compared to lightweight baselines, suggesting that further optimization is required for real-time deployment on resource-constrained edge devices. Third, the effectiveness of Shape-IoU is validated on the current dataset; however, its generalization to other object detection tasks with different shape distributions requires further investigation. Furthermore, several challenging failure cases still exist in practical PV inspection scenarios. For example, extremely small-scale defects, weak thermal anomalies, or subtle surface contamination (e.g., small dust spots or slight stains) may not produce sufficiently distinguishable responses in either visible or infrared modalities. Under such conditions, the complementary information between modalities becomes limited, reducing the effectiveness of cross-modal fusion. Furthermore, when both modalities simultaneously suffer from weak contrast, thermal diffusion, or background interference, the model may still exhibit missed detections or unstable localization performance. These challenges indicate that dual-modality fusion alone cannot completely eliminate all ambiguity in complex industrial environments, and more discriminative feature representation and fine-grained anomaly modeling strategies are still required.

Future work will focus on three directions. First, more advanced cross-modal alignment strategies, such as deformable alignment or Transformer-based interaction mechanisms, can be explored to further improve feature consistency. Second, lightweight model design can be further optimized to enhance real-time performance without sacrificing accuracy. Third, expanding the dataset scale and diversity, as well as evaluating the model on more challenging real-world scenarios, will help to improve robustness and generalization capability, paving the way for more reliable and practical PV inspection systems. In addition, although the proposed GMD-YOLO framework is developed for photovoltaic fault detection, its architecture is not limited to PV scenarios. Since the proposed MSPPD, GAFusion, and Detect-DEhead modules are designed for multi-scale contextual modeling, adaptive cross-modal interaction, and dynamic feature representation, the framework can potentially be extended to other dual-modality industrial inspection tasks, such as power line inspection, pipeline monitoring, industrial crack detection, and UAV-based thermal anomaly analysis. In particular, tasks involving complementary visible–infrared information and complex environmental interference may benefit from the proposed adaptive fusion and geometry-aware localization mechanisms.

## 5. Conclusions

This paper addressed the limited robustness of single-modality methods in PV module fault detection under complex environments. We proposed a dual-modality visible–infrared fusion detection framework based on YOLO11. Specifically, the MSPPD, GAFusion, and Detect-DEhead modules were introduced into the feature extraction, cross-modality fusion, and detection head stages, respectively. This design effectively exploits complementary multimodal information, enhancing detection for multiple fault types, including hotspots, diode short circuits, occlusions, and shadows. Furthermore, the Shape-IoU loss function was incorporated to improve bounding box localization accuracy and convergence.

Ablation experiments conducted on the YOLO11 dual-modality baseline demonstrate that each proposed module contributes to performance improvement. When all three modules are jointly applied, the model achieves the best overall performance, with mAP@0.5 improving from 86.7% to 88.1% (+1.4%). Meanwhile, the number of parameters and computational complexity are reduced by 13.95% and 17.95%, respectively, achieving a favorable balance between detection accuracy and efficiency. Furthermore, a comparative analysis of different IoU-based loss functions shows that, without increasing model complexity, Shape-IoU improves mAP@0.5 from 88.1% to 88.4% and recall from 78.5% to 84.9%, demonstrating its effectiveness in enhancing localization accuracy and recall performance.

Experimental results on the publicly available FLIR Thermal dataset further validate the generalization capability of the proposed method in complex scenarios. Specifically, precision improves from 78.2% to 80.4% (+2.2 percentage points), recall increases from 61.9% to 63.5% (+1.6 percentage points), and mAP@0.5 rises from 69.8% to 72.5% (+2.7 percentage points). At the same time, the model parameters are reduced to 3.7 M, GFLOPs decrease to 9.36, and the model size is reduced to 7.9 MB, confirming that the proposed method achieves improved detection performance while maintaining a lightweight design.

Despite these improvements, several limitations remain. For instance, cross-modality alignment between infrared and visible data remains suboptimal under extreme environmental conditions. In addition, the trade-off between further model lightweighting and real-time performance requires further optimization. Moreover, the generalization capability of Shape-IoU across different object types and more complex scenarios requires further validation.

In addition, the current framework is designed for single-frame detection and does not explicitly model temporal information. In real UAV-based photovoltaic inspection scenarios, sequential flight frames contain rich temporal consistency and motion-aware cues. Future work will explore temporal fusion strategies based on sequential UAV images, such as temporal attention mechanisms and video-level feature aggregation, to further enhance robustness in dynamic environments and reduce false detections caused by transient noise, viewpoint variations, and illumination fluctuations. These directions are expected to further improve the adaptability and practical deployment capability of the proposed method in real-world photovoltaic inspection systems.

## Figures and Tables

**Table 1:** Experimental results of YOLO11-RGBT at different fusion stages.

YOLO11n-RGBT	P/%	R/%	mAP50/%	Params/10^6^	GFLOPs/10^9^	Model Size/MB	FPS
Early fusion	80.6	77.9	86.1	2.6	6.51	5.5	104.15
Mid fusion	77.3	77.9	85.9	3.8	9.52	8.0	81.46
Mid-to-late fusion	80.4	81.2	86.7	4.3	11.42	9.1	71.85
Late fusion	81.6	78.7	86.3	5.2	12.56	10.8	61.82

**Table 2:** AP comparison of different fault types between single-modality infrared and dual-modality (infrared and visible) models.

Modol	Datasets	HotSpot	Occlusion	Diode Short Circuit	Shadow	mAP50/%
YOLO11n	IR	93.1	78.2	90.5	77.2	84.7
YOLO11n-fusion	IR + RGB	91.8	85.5	89.0	80.2	86.7

Note: YOLO11n-fusion is YOLO11n mid-to-late fusion.

**Table 3:** Ablation study on different module configurations.

Test	A	B	C	P/%	R/%	mAP50/%	Params/10^6^	GFLOPs/10^9^	Model Size/MB	FPS
1				80.4	81.2	86.7	4.3	11.42	9.1	71.85
2	√			80.3	82.0	87.0	4.2	10.45	8.8	64.21
3		√		79.7	81.6	86.9	4.0	11.16	8.5	67.10
4			√	83.0	80.4	87.1	4.2	10.12	8.8	62.29
5	√	√		79.8	80.6	87.1	3.9	10.19	8.2	63.75
6		√	√	84.8	78.9	87.7	3.8	9.86	8.4	60.46
7	√		√	81.8	77.9	87.3	4.0	9.63	8.5	62.70
8	√	√	√	83.2	78.5	88.1	3.7	9.37	7.9	59.55

Note: A is the GAFusion module; B is the MSPPD module; C is the Detect-DEhead module. A check mark (√) indicates that the corresponding module is introduced to replace or enhance the related component in the baseline model.1 is the mid-to-late fusion YOLOv11n model; 2 is the mid-to-late fusion YOLOv11n + GAFusion model; 3 is the mid-to-late fusion YOLOv11n + MSPPD model; 4 is the mid-to-late fusion YOLOv11n + Detect-DEhead model; 5 is the mid-to-late fusion YOLOv11n + GAFusion + MSPPD model; 6 is the mid-to-late fusion YOLOv11n + MSPPD + Detect-DEhead model; 7 is the mid-to-late fusion YOLOv11n + GAFusion + Detect-DEhead model; 8 is the mid-to-late fusion YOLOv11n + GAFusion + MSPPD + Detect-DEhead model.

**Table 4:** Comparison of different IoU-based loss functions.

GMD-YOLO (Loss Function)	P/%	R/%	mAP50/%
CIoU	83.2	78.5	88.1
Shape-IoU	78.5	84.9	88.4

**Table 5:** Experimental results of different YOLO versions under dual-modality mid-to-late fusion.

Mid-to-Late Fusion	P/%	R/%	mAP50/%	Params/10^6^	GFLOPs/10^9^	Model Size/MB	FPS
YOLOv5	81.5	79.1	86.5	4.5	14.99	9.5	87.02
YOLOv6	79.5	80.9	85.8	8.2	21.24	16.7	95.57
YOLOv7-tiny	82.2	77.9	83.1	13.9	42.96	28.3	75.83
YOLOv8	81.7	77.9	86.2	5.2	16.67	10.9	85.65
YOLOv9t	82.9	77.7	86.2	3.4	15.93	8.1	29.96
YOLOv10	81.1	79.3	85.6	8.3	26.83	17.3	66.36
YOLO11	80.4	81.2	86.7	4.3	11.42	9.1	71.85
YOLO12	79.9	72.3	84.7	4.2	11.45	9.2	39.79
YOLO13	81.9	76.5	85.0	6.7	15.66	14.3	33.30
GMD-YOLO	83.2	78.5	88.1	3.7	9.37	7.9	59.55

**Table 6:** Comparison of detection performance under different illumination conditions.

Conditions	Model	P/%	R/%	mAP50/%	Params/10^6^	GFLOPs/10^9^	Model Size/MB	FPS
overcast	YOLO11	82.5	76.1	85.0	3.7	9.37	7.9	71.85
	GMD-YOLO	82.6	74.4	85.4	3.7	9.37	7.9	59.55
low-light	YOLO11	79.4	78.4	85.5	3.7	9.37	7.9	71.85
	GMD-YOLO	81.0	78.4	85.7	3.7	9.37	7.9	59.55

**Table 7:** Comparison between the baseline and improved models on the FLIR Thermal dataset.

Mid-to-Late Fusion	P/%	R/%	mAP50/%	Params/10^6^	GFLOPs/10^9^	Model Size/MB	FPS
YOLO11n	78.2	61.9	69.8	4.3	11.42	9.1	74.34
GMD-YOLO	80.4	63.5	72.5	3.7	9.36	7.9	60.12

## Data Availability

The self-constructed dataset generated and used in this study is not publicly available due to ongoing research use within our group. The experimental results reported from public datasets were generated by running our own model on those datasets. All derived data supporting the findings of this study are available from the corresponding author upon reasonable request.
